# Supplementary material for: Excess maternal mortality in Brazil: Regional inequalities and trajectories during the COVID-19 epidemic
Source: PLoS One. 2022 Oct 20;17(10):e0275333. doi: 10.1371/journal.pone.0275333 (PMC9584504; doi:10.1371/journal.pone.0275333)
Supplement: S2 Table — (DOCX) [file pone.0275333.s002.docx]

**Table S2.** Description of the five main causes of death from the blocks containing the most information on maternal causes of death in chapter XV of International Statistical Classification of Diseases and Related Health Problems (ICD) 10th Revision, according five consecutive trimonthly periods, March 2020 to May 2021, Brazil.

| ICD code | Description | March-May/2020 | June-August/2020 | September-November/2020 | December/2020-February/2021 | March-May/2021 |
| --- | --- | --- | --- | --- | --- | --- |
| **O85-O99**  **Complications predominantly related to the puerperium and other obstetric conditions, not elsewhere classified** | | | | | | |
| O98.5 | Other viral diseases complicating pregnancy, childbirth and the puerperium | 135 | 191 | 73 | 182 | 767 |
|  |  |  |  |  |  |  |
| O99.4 | Diseases of the circulatory system complicating pregnancy, childbirth and the puerperium | 32 | 24 | 30 | 23 | 25 |
|  |  |  |  |  |  |  |
| O95 | Obstetric death of unspecified cause | 27 | 20 | 20 | 16 | 21 |
|  |  |  |  |  |  |  |
| O99.8 | Other specified diseases and conditions complicating pregnancy, childbirth and the puerperium | 30 | 16 | 14 | 15 | 19 |
|  |  |  |  |  |  |  |
| O99.5 | Diseases of the respiratory system complicating pregnancy, childbirth and the puerperium | 24 | 13 | 6 | 7 | 20 |
| **O10-O16**  **Oedema, proteinuria and hypertensive disorders in pregnancy, childbirth and the puerperium** | | | | | | |
| O14.1 | Severe pre-eclampsia | 28 | 19 | 24 | 23 | 25 |
|  |  |  |  |  |  |  |
| O15.9 | Eclampsia, unspecified as to time period | 16 | 14 | 10 | 19 | 12 |
|  |  |  |  |  |  |  |
| O14.9 | Pre-eclampsia, unspecified | 9 | 16 | 9 | 14 | 22 |
|  |  |  |  |  |  |  |
| O15.0 | Eclampsia in pregnancy | 9 | 12 | 10 | 9 | 16 |
|  |  |  |  |  |  |  |
| O15.2 | Eclampsia in the puerperium | 8 | 6 | 6 | 6 | 4 |
|  | | | | | | |
|  | | | | | | |
| ICD code | Description | March-May/2020 | June-August/2020 | September-November/2020 | December/2020-February/2021 | March-May/2021 |
|  |  |  |  |  |  |  |
| **O20-O29, 60-O63, O67-O71, O73-O75, O81-O84**  **Other complications of labour and delivery** | | | | | | |
|  |  |  |  |  |  |  |
| O62.2 | Other uterine inertia | 12 | 14 | 7 | 8 | 16 |
|  |  |  |  |  |  |  |
| O75.9 | Complication of labour and delivery, unspecified | 3 | 5 | 3 | 5 | 10 |
|  |  |  |  |  |  |  |
| O26.6 | Liver disorders in pregnancy, childbirth and the puerperium | 5 | 6 | 7 | 3 | 3 |
|  |  |  |  |  |  |  |
| O23.4 | Infections of other parts of urinary tract in pregnancy | 5 | 2 | 2 | - | 3 |
|  |  |  |  |  |  |  |
| O24.4 | Diabetes mellitus arising in pregnancy | - | 3 | - | 2 | 2 |
|  |  |  |  |  |  |  |
